# A Tale of Two Sites: Lessons on Leadership from the Implementation of a Long-term Care Delivery Model (CDM) in Western Canada

**DOI:** 10.3390/healthcare4010003

**Published:** 2016-01-04

**Authors:** Denise Cloutier, Amy Cox, Ruth Kampen, Karen Kobayashi, Heather Cook, Deanne Taylor, Gina Gaspard

**Affiliations:** 1 Department of Geography and Centre on Aging, University of Victoria, Victoria, BC V8W 2Y2, Canada; 2 Department of Sociology and Centre on Aging, University of Victoria, Victoria, BC V8W 3P5, Canada; amygc@uvic.ca (A.C.); rkampen@uvic.ca (R.K.); kmkobay@uvic.ca (K.K.); 3 Interior Health Authority, Kelowna, BC V1Y 4N7, Canada; Heather.Cook@interiorhealth.ca (H.C.); Deanne.Taylor@interiorhealth.ca (D.T.); 4 First Nations Health Authority, Vancouver, BC V6C 1A1, Canada; Gina.Gaspard@fnha.ca

**Keywords:** care delivery model, leadership, direct care staff, long-term care, team work, change management

## Abstract

Residential, long-term care serves vulnerable older adults in a facility-based environment. A new care delivery model (CDM) designed to promote more equitable care for residents was implemented in a health region in Western Canada. Leaders and managers faced challenges in implementing this model alongside other concurrent changes. This paper explores the question: How did leadership style influence team functioning with the implementation of the CDM? Qualitative data from interviews with leadership personnel (directors and managers, residential care coordinators and clinical nurse educators), and direct care staff (registered nurses, licensed practical nurses, health care aides, and allied health therapists), working in two different facilities comprise the main sources of data for this study. The findings reveal that leaders with a servant leadership style were better able to create and sustain the conditions to support successful model implementation and higher team functioning, compared to a facility in which the leadership style was less inclusive and proactive, and more resistant to the change. Consequently, staff at the second facility experienced a greater sense of overload with the implementation of the CDM. This study concludes that strong leadership is key to facilitating team work and job satisfaction in a context of change.

## 1. Introduction

Facility-based long-term care (e.g., residential care settings and nursing homes) occupies a critical place in the health care continuum; maintaining and supporting frail older adults and their families on an ongoing basis [1,2]. As individuals live longer, care providers (both formal and informal), face the challenge of providing care to clients who are increasingly complex; having multiple chronic conditions, including dementia and other illnesses that affect cognitive functioning and behaviour [3,4]. These conditions of increased client complexity alongside cost constraints pose extraordinary challenges to governments and health planning organizations in the provision of around the clock, person-centred, cost-effective care [2,3,4].

In this environment, new programs, policies and models of care delivery are continuously being developed and implemented in a climate of change management [2,4]. Strong and effective leadership is key to navigating these changes and supporting care teams [5]. In residential long-term care (LTC), registered nurses often occupy the top, executive positions as managers and administrators [6]. These roles demand sophisticated business knowledge as well as clinical competency [6].

A new care delivery model (CDM) designed to promote more equitable care for residents regardless of where they lived and received care was implemented in the long-term care environment in a Western Canadian health region. The primary goal of the CDM was to standardize the care provided to every resident in facility-based long-term care (LTC) in the region. Leaders and managers were challenged to implement this model alongside other concurrent changes which are discussed. In this paper we aimed to understand how the CDM implementation, a significant change initiative, instituted in all care facilities in the region was influenced by leadership and experienced by direct care staff.The central research question for this paper therefore wasis: “How did leadership style and approach influence team functioning with the implementation of the CDM?”

This paper is part of a larger three-year, mixed methods (qualitative and quantitative) study that took place in three residential care facilities, owned and operated by the health region. Relative to our central question, and to highlight CDM implementation experiences through the lenses of leadership and team functioning, we focus in this paper on two of the three facilities (Facility A and B) in which the larger evaluation study was conducted. These two sites had different experiences with model implementation, notably from highly positive (Facility A) to more challenging (Facility B). Our rationale for excluding the third site (Facility C), from this analysis was that after interviews had been conducted, it was observed that Facility C had been moving towards the CDM model more gradually since 2006 rather than from 2011; and hence the specific impact of the CDM change initiative at this site was more difficult to isolate and evaluate.

The main data for this qualitative analysis are drawn frominterviews with key administrative personnel, that is local leadership staff (*i.e.*, Residential Care Coordinators (RCCs) and clinical nurse educators (CNEs); and direct care staff (*i.e.*, RNs, LPNs and health care aides (HCAs), unit clerks and allied health therapists (recreation, physiotherapists, occupational therapists and social workers), who worked in the two different residential care facilities in a populous health region in Western Canada.

The remainder of this paper is organized into four sections: a research context section that discusses the Care Delivery Model (CDM) and the conditions prior to model implementation. We then move to a discussion of change management, drawing on the work of experts and considering the qualities of leaders and leadership problems (both technical and adaptive), in the context of a transformative change initiative. The research context section is followed by the methodology, findings, and finally, a combined discussion and conclusion section in which the specific and broader findings, and study limitations are discussed.

## 2. The Research Context

The facility-based residential long-term care sector typically provides both publicly (subsidized) and privately funded (non-subsidized) care on a 24/7 basis in a protective, supportive environment for people with complex care needs who are no longer able to live independently in the community [1,2]. Residential care (RC) programs employ both regulated (e.g., registered nurses (RNs), licensed practical nurses (LPNs), physical therapists, occupational therapists, recreation therapists, dietitians) and unregulated staff (health care assistants (HCAs) who assist with social and medical needs related to daily living (*i.e.*, pain and symptom management, rehabilitation, assistance with dressing, bathing, feeding, toileting), and recreational activities (*i.e.*, socializing, crafts, games). Over the past several decades, financial pressures and downward shifts in the size of the trained workforce, and ongoing change initiatives have challenged leaders in the provision of care to increasingly frail older persons [2,3,5,7,8,9,10].

### 2.1. The Care Delivery Model (CDM)

Initiated in 2011, the CDM represents one example of a transformative change model of health service delivery designed to improve equity in service provision by promoting more “hands- on care” to each resident. This goal was enacted through three inter-related initiatives: (1) strategies to change the staff mix ratio for direct care staff, that is, reducing RNs, in order to increase the amount of hands-on care provided by LPNs and HCAs; (2) setting a target standard of 3.0 h of direct care per resident per day; and (3) standardizing the funding methodology across public (subsidized), and private (non-subsidized) facilities in the health region, to ensure that regardless of where they lived, residents would receive the same level of care.

The focus in this paper is on facility level leadership and direct care staff. In the health region in which the research was conducted, the leadership team was comprised of: managers, directors, CNEs and RCCs. Direct care to residents was predominantly provided by RNs, LPNs, HCAs and allied health therapists. CNEs and RCCs were the first point of contact for staff to access managers. CNEs and RCCs are usually RNs by training (having advanced nursing skills, sometimes with specific leadership training), and they provide face-to-face, clinical leadership and act as sounding boards for medical staff and family members should issues arise.

With model implementation, the direct care roles of LPNs and HCAs continued to focus on the provision of clinical care and meeting daily care needs, while the role of RNs changed more to one of clinical oversight, with greater responsibility for coordinating and mentoring other direct care staff, rather than *providing* as much direct care themselves to residents. Reducing the number of RNs (who received higher wages), allowed the leadership to hire more LPNs and HCAs to provide this hands-on care to residents. In other words, with model implementation a larger group of staff (*i.e.*, interdisciplinary team) could be called upon to address the complex care needs of residents, but the skills mixes and composition of the team were altered.

### 2.2. Conditions around the Time of Model Implementation

It must be noted that this was a study that was conducted in a real-world setting which meant that many conditions, and concurrent changes (discussed below) could not be controlled. For example, the prior history of facilities in terms of values, leadership and team dynamics also played a role in shaping how the CDM was received and enacted.

Initially, it must be stated that all interview participants, both managers and direct care staff at both facilities, were united in their primary concern for the care of residents. The CDM was an example of a transformative change model within the residential care sector which significantly altered staff composition/staff mix, and therefore team functioning. While there were many other notable, concurrent changes in the system at the time of the CDM, two are parjticularly important. The first was a recently mandated resident assessment tool (MDS-RAI-RC), and the second was the “single certification” initiative. The MDS tool altered regular practice by requiring direct care staff to complete a standardized assessment on residents at regular intervals (e.g., at intake, quarterly and annually, or upon a change in resident status). This assessment tool was often perceived as an “add on” to existing practice and information gathering.

In this unionized environment, single certification was a collective bargaining initiative that allowed HCAs initially, followed by LPNs and then RNs, to have greater job portability to relocate their position without losing seniority or benefits. This initiative had a differential effect on staff in care facilities depending, to a substantial degree, on the current workforce composition and leadership at the time it was introduced. For some facilities, the significant movement of workers as a result of single certification, presented unique challenges for leadership, with respect to the hiring and training of new staff, and also with regard to team cohesion, as in the case of the CDM. However, all displaced employees working in any facility at the time of CDM implementation, were given options for employment within the health region, or where appropriate, offered retirement packages.

### 2.3. Change Management and Qualities of Leaders

Historically, models of care delivery in health and related fields emphasized delivering care to “patients” in hierarchical, top-down, often system-centred ways by solo-practitioners working in isolation from each other [11]. However, over the last few decades, there has been an increasing emphasis on the importance of changing the culture of care by delivering care in more collaborative, and person-centred ways through skilled, interdisciplinary teams and multi-level participation [12,13,14,15,16,17,18,19,20,21]. Today, the Canadian health system reflects a perpetually shifting landscape in terms of the tendency towards change whether minor or transformative [11,19]. Consequently, health care leaders must constantly adapt and find ways to move forward, ideally in innovative, positive and productive ways [20,21]. Standing still is not an option, and there are many models for care delivery.

These conditions and realities are studied within the evolving field of change management. Simply defined, change management is a systematic approach to dealing with change, both from the perspective of an organization, and the individual, that focuses on adapting to change, controlling change, or effecting change [15,17,21]. Concepts of participative leadership, empowerment and systems thinking are important in this arena [21,22,23]. Campbell [15] advises health care managers to draw on the theories of Kotter and Cohen [21]; and Bridges [22] in leading through times of transformative change. Kotter and Cohen [21] offer an eight-step process for change management: (1) create a sense of urgency; (2) build guiding teams; (3) get the vision right; (4) communicate for buy-in; (5) enable action; (6) create short-term wins; (7) don’t let up; and (8) make it stick. They divide their steps into three phases called: creating the climate for change (1–3), engaging and enabling the whole organization (4–6) and implementing and sustaining change (7–8) [21,22,23]. Bridges [22], on the other hand distinguishes between “changes” which are situational and “transitions” that are psychological —the latter requiring internal re-patternings, *i.e.*, adopting a new way of working. He maintains that people get caught in a “neutral zone” vacillating between the old (endings) and new (beginnings) systems, and it is the job of leaders to move employees out of a sense of loss over how things worked in the past, towards a sense of life as a journey going forward. The successful implementation of new strategies and new models depends upon *how* things are brought into play and the human resource strategy is critical to overall success [24]. In a survey of nurse leaders working in hospital settings, education and public health, the top three competencies for leaders in order of reported importance were: (1) Building effective teams; (2) Communicating vision and strategies internally; and (3) Translating visions into strategies [5]. All of these ideas are interwoven and common elements in the change management literature.

Within the literature on leadership styles, Trastek *et al.* [24] argue that “servant leadership” is one of the best forms of leadership for health care because it focuses on the strengths, contributions and development of trust within the team, and serving the needs of patients. Servant leaders lead in a “side-by-side” rather than top down fashion exemplifying qualities of integrity and professionalism, and they are skilled at visioning, strategizing, tactics, aligning resources, inspiring others, and executing plans [25,26,27]. This style of leadership is different from a more “transactional,” command and control type of top-down hierarchical leadership that has characterized health care in the past [25,26,27]. Servant (or adaptive) leaders operate according to collective rather than individual interests. Through their deep commitment to the growth of people (employees and recipients of care), they may be considered to be “heart-centered”, managing by demonstrating qualities of empathy, awareness, persuasion, conceptualization, foresight, and stewardship [23]. They lead by establishing systems of shared values and by creating opportunities to support empowerment and community-building. Under servant leaders, workers may be intrinsically motivated to change by having these shared values. Finally, being concerned with the way things work “on the ground”, these leaders are reality-focused rather than only being “big picture” or goal-focused [28].

Not surprisingly, a key task of leadership is problem-solving with said problems lying on a continuum between known problems with known solutions (technical problems) and difficult to define problems with unknown solutions (adaptive challenges) [28,29,30]. Technical problems may be very complex and critically important like replacing a faulty heart valve during cardiac surgery, but they have a known solution that is addressed by current know-how and resolved through the application of authoritative expertise in relation to an organization’s current structures, procedures, and culture [31]. Technical problems often fit well with a transactional command and control, top-down type of leadership where there is interest: in getting people to follow a prescribed pathway, where rapid decisions are needed, and where there may be resistance to looking for solutions outside the “normal” comfort zone [30]. With adaptive challenges the exact nature of the problem is often difficult to identify and the solutions are often diffuse and multi-pronged requiring creativity and experimentation [25]. Because of their complexity, adaptive challenges often necessitate changes in people’s priorities, beliefs, habits, and loyalties [30,31]. According to Heifitz, Grashow and Linsky [30] “the most common failure of leadership is produced by treating adaptive challenges as if they were technical problems” (p. 19).

To illustrate how adaptive and technical problems and solutions can dovetail, Corazzini *et al.* [28] take the issue of improving quality of life for residents through building social connections. This is not a technical problem and yet it could be solved by a technical solution such as making straightforward changes to how tasks are scheduled to encourage more social time for residents. Conversely, fostering social connections could also be viewed as an adaptive challenge in which the emphasis would be on the importance of supporting relationship-building between staff and residents as a core value within an institution (*i.e.*, the movement toward more patient or person-centred care) which requires considering how an organization operates, what it values, how staff work together, and how jobs are structured to begin with, thereby entailing much larger considerations than simply emphasizing the (technical) performance of tasks (e.g., number of baths, number of congregate meals in a week, *etc.*) as a means of improving social connections and quality of life.

## 3. Methodology

**Figure 1:**
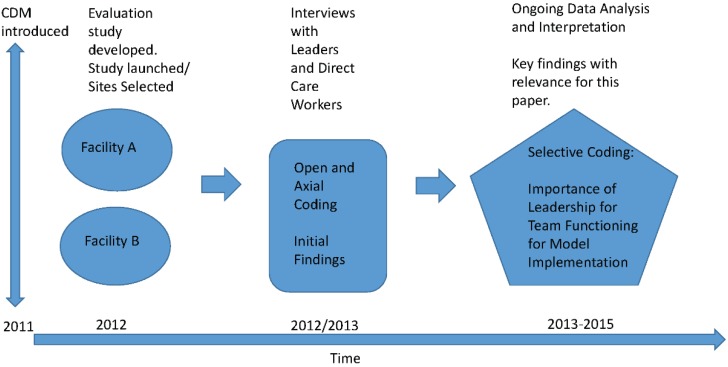
CMD implementation evaluation study.

Within the larger three-year study to evaluate the implementation of a care delivery model, this paper represents a qualitative analysis drawing on interviews with leaders, and direct care staff working in two different residential care facilities. These facilities were purposively selected on the basis of geographic representation, facility size (*i.e.*, medium-sized with 90–120 residents), and CDM implementation in 2011. A study flow chart is provided in Figure 1.

In understanding the impact of the CDM in the study area, it is instructive to consider how the staff mix changed both pre- and post- implementation. The biggest changes were in the decrease in direct care provided by RNs as they took on roles of clinical oversight, and the increase in hands-on care by LPNs. Results from the quantitative phase of the study at Facility A indicated that RNs were reduced from 15% to 10% of the direct care staff, LPNs increased from 9% to 19%, and HCAs went from 76% to 71% with model implementation. B experienced larger fluxes in staff mix. Notably, the percentage of RNs decreased from 24% to 8%, LPNs increased from 6% to 24%, and HCAs remaining fairly stable, representing from 70% to 68% of the staff complement. It is also important to note that there were more part-time RNs working at Facility B.

Hours of care also changed among the three practice groups. At Facility A, the *amount* of care per resident, provided by RNs decreased slightly by 0.44 h to 0.32 h on average, while LPN hours doubled from 0.25 h to 0.57 h, with HCAs providing the bulk of care at 2.16 h, which was fairly stable both before and after CDM implementation. At Facility B, RN hours of care decreased by 2/3 from 0.65 to 0.24, while LPN hours increased by more than a factor of 4, from 0.16 to 0.73, and HCA hours also increased from 1.87 to 2.04 h. In summing these hours, it is notable that the total hours of direct care to residents remained essentially the same. The difference was in who was providing the care. Hands-on care to residents (provided largely by HCAs, was either relatively stable (Facility A), or increased slightly (Facility B). Further, at Facility B, most of the leadership team had been in their positions for less than five years, whereas staff at Facility A had occupied their roles for considerably longer, ranging from 10 years to more than 20 years for the current manager.

### 3.1. Data Collection

Research team members visited each facility to introduce the study to staff and to recruit participants. Pamphlets explaining the study were left at each location for staff to review. Additionally, research team members communicated with facility leadership teams to help promote the study. Prior to conducting the interviews, ethics approval was received jointly from the Human Subjects Ethics Review Committee of the university from where the academic researchers were located and the health region’s ethics approval committee. Qualitative interview data were undertaken by authors AC and RK and collected primarily through one-on-one face-to-face interviews, as well as in small group interviews in some instances, with 2–6 participants. These group interviews were organized by staff role to reduce any possible power imbalance that could arise, and to ensure that participants felt able to voice their opinions confidentially. Individuals were voluntary participants at these sessions, but their presence was also based on scheduling and availability. A semi-structured interview guide, with allowances for open-ended discussion from participants was used. Leadership team interviews were undertaken with RCCs, CNEs, Managers, and Directors. Interviews were also conducted with direct care nursing staff (RNs, LPNs and HCAs) as well as allied health staff, including Recreation Therapists and Physiotherapists. The interview guides included questions about the impact of the model implementation, definitions of quality of care, other concurrent changes in the facility, communication between different groups, teamwork, and job satisfaction.

Interviews occurred at two time points (summer 2012-Round 1, and summer 2013-Round 2). Most leadership members were interviewed twice (*i.e.*, once at each of these time points), while direct care staff were predominantly only interviewed at one point in time, due to attrition, movement of staff within the system, and scheduling conflicts. During Round 1, 17 interviews with 30 participants were conducted for Facility A, and 16 interviews with 22 participants for Facility B. For Round 2, a total of 25 interviews were conducted with 33 participants for Facility A and 25 interviews with 33 participants for Facility B. The majority of the interviews were conducted in person, with only five completed over the telephone. Each interview was audiotaped, and transcribed by a professional transcriptionist.

### 3.2. Data Interpretation

All transcripts were entered into NVivo 10, a software program that was used to “code” (organize and retrieve) excerpts of the data for the qualitative analysis [32]. Interviews were read and coded by the interviewers to develop an initial coding scheme. Next, monthly meetings of the entire research team (comprised of academics and health region personnel), occurred to validate the initial scheme and shape the emergence of additional selective and focused codes or themes [33]. Interpretive validity and rigor were also supported by regular meetings with an Advisory Panel, established at the outset of the project and comprised of leadership team members such as managers, directors, residential care coordinators, social workers, recreation therapists, an RAI-MDS analyst, and physicians with experience working in the health region, but not at the specific study facilities. The Advisory Panel (AP) met seven times over the course of the three-year study and contributed to data interpretation, validation and oversight for the project.

In addition, an analytical and methodological log (audit trail) was developed and used throughout the project to support rigor and to capture and record analytical decisions as the project unfolded [33,34,35]. The findings from this paper are based on the data collected and coded under seven themes arising from these processes: leadership, vision and values, team work, empowerment, feeling supported, lack of control, and barriers to change. These processes supported consensus-building and helped guard against fixed or biased conclusions on the part of academic team members while also challenging biases and assumptions from practice team members [34,35].

## 4. Findings

This section is presented in two parts. Since model implementation experiences were unique at each facility, part one reflects the thematic results emerging from Facility A interviews, while part two offers a contrasting look at Facility B. Facility A, experienced CDM implementation in a fairly seamless way, while Facility B faced greater challenges. We use Kotter and Cohen’s [21] three broad phases: creating the climate for change, engaging and enabling the whole organization, and implementing and sustaining change, and Bridge’s [22] notion of the neutral zone to organize and contextualize the findings, and highlight experiences with model implementation.

### 4.1. Facility A

At Facility A, creating a climate for change involved a stage of readiness-building that began well before CDM implementation:

*I’m trying to think*; *maybe nine or ten years ago... and so again in keeping with the idea of empowering people and servant leadership*, *you want to be inclusive for all the stakeholders…And*, *we met with some LPNs. Well*, *we put out an invitation to anyone who wanted to be a part of the committee of putting together this new model*, *and what it would look like and how we would kind of divvy up responsibilities and tasks and leadership*, *and so on and so forth. And so*, *[with the CDM]….we got together and there was…It starts off of course as a surprise*, *and an upset to people because of course people feel in jeopardy that they’re going to lose their job.*

*And*, *so then*, *what we did is we talked to each of the individual RNs together because there was going to be a reduction in the number of RNs*, *but we talked to them individually to find out what are your dreams?*, *and what are your wishes? And*, *is your dream to be here*, *or is this an opportunity and kind of a segue into something that you really want to do?*

*So*, *having the staff who are going through the transition*, *the RNs and the LPNs*, *having them involved every step of the way was so important. What do you think this is gonna look like and how can we support you?…The conversation was always what can I do to support you? And*, *that is a big difference on this team.…And*, *they were very much a part of all the conversations ahead of time.*

*(Nurse Leadership*, *Facility A)*

Recognizing that the RNs who provided direct care were among those staff members with the greatest role changes through the CDM, leadership sought to alleviate their concerns, worries and anxieties by engaging them in discussions about how the new model would be rolled out, and how they saw themselves fitting within the new way of working. By asking them about their dreams and wishes at the outset, they modeled inclusiveness and minimized the threats brought on by potential job losses and restructuring. If individuals did not see themselves fitting in to the new CDM model reality, they had the opportunity to move to a different organization or go back to acute care, for example.

Facility A management emphasized working with the entire team in a servant style of leadership to develop solutions:

*…everybody’s role is recognized right—from the housekeeper to the person who maintains the physical plant to the casual visitor*; *that everybody has a place and everybody has an opportunity to contribute to the quality of life of the person whose home it is right?*

*(Allied health worker*, *Facility A)*

Health care aides expressed their view that their jobs were challenging but that management (and family members) supported and appreciated their efforts:

***HCA2:*** *I think we’ve got a very good working environment here.*

***HCA1:*** *We do.*

***HCA2:*** *Where we are recognized both by the resident*, *even the families…*

***HCA1:*** *Yeah.*

***HCA2:*** *We do have families who would come and say “Where have you been? We missed you. We are so grateful for what you are doing for our dear ones.” And then also we have a very good management that I have to mention that because actually it’s like you feel that you are in the right place and what you are doing everybody can see it and organize it and that’s very important. When you’re feeling motivated…When I come in the morning and I find maybe one of our managers I find maybe a statement made in the newspapers on appreciating people and then they say you are the superb team*, *these kind of things*, *you just…*

***HCA1:*** *Yeah.*

***HCA2:*** *You just went oh…I wish I would be able to say this to other people because I say we are not perfect*, *but somebody addressing an issue saying you are the best. You are the best. Or maybe you are the A+ team.*

***HCA:*** *Yeah*, *yeah.*

***HCA2:*** *You come and you find those kinds of messages there*, *and now you see it doesn’t mean we are perfect. Everything is so good*, *there is nothing to say*, *but so to see somebody who can see only the…How do they say they see the full glass*, *not the half empty glass. It is not so easy*, *but we have very good management that can see the half full glass.*

*(health care aides*, *Facility A)*

At Facility A, staff received a copy of the facility vision when they were hired. It was a living, real document for them. Having a common vision, purpose and shared goals facilitated teamwork and reduced negative behaviours associated with working in an environment often dominated by hierarchies. And, many transcripts with participants from Facility A, revealed that staff felt supported even though they also recognized the constraints on local resources:

*I hear from people that one of the differences between our facility and a lot of the others is they [here] just feel supported*, *and that can mean that they have a voice if they feel frustrated*; *that if they disagree they can even voice that*, *but at the same time you know we all have the same lack of resources.…It’s not like our facility has more resources than anyone else*, *but people*, *I think they feel supported by the leadership. We recognize where we have struggles with resources.*

*(Nurse Leadership*, *Facility A)*

These quotes from leadership and allied health staff reflect the second phase in Kotter and Cohen’s [21] schematic for change management: engaging and enabling the whole organization in the change. Through a high level of commitment to feedback, communication and consultation with staff, a common vision for going forward was established tied to joint values of ensuring quality of care to residents, and being valued members of the care team.

Facility A leadership exemplified compassion and caring. Staff felt heard about their concerns, but also felt they could discuss issues as they arose. Leadership spoke about how they wanted their offices to be perceived as a “safe space” for dialogue, while staff said, “we know she has our back”, an idea that reinforced the feeling that leaders and staff were in it together, thus strengthening a sense of community among team members, and cultivating conditions for safe, positive and empowering relationship-building among staff persons. At Facility A, the RCC had a “we’ll get it done together,” pitch-in attitude that ended up flattening the leadership hierarchy, and the kind of side-by-side relationships consistent with a servant leadership approach:

*I’ve heard [in] a lot of places it’s a definite hierarchy*, *but here it’s not—we’re just a team. (LPN*, *Facility A).*

In terms of the third phase of implementing and sustaining change, the leadership were engaged with direct care staff through a range of additional, formalized administrative activities (e.g., development of support plans, performance reviews and goal setting exercises, and weekly check in meetings), all designed to help individual staff to establish a sense of their own goals within the environment. As a consequence of these approaches and mechanisms, staff at Facility A, spoke positively about the CDM and conveyed a strong sense of working together as members of a high functioning team. The research team interviewers also echoed their perceptions of staff interviews at Facility A as largely “positive” and “uplifting”. Helping staff to work to their highest potential is another aspect of servant leadership exemplified in the following excerpt:

*…and*, *there’s just a lot of hand holding*, *supporting staff where they were at*, *but at the same time moving us forward little*, *by little*, *by little…helping all [staff] to develop their roles*, *and they had a huge say in what that was gonna look like. I mean within the boundaries of what we were required to do.*

*(Manager*, *Facility A)*

The above quote also reflects the importance of realistic goals and managing staff expectations through slower, incremental processes. Team functioning at Facility A also appeared to be linked to a kind of fluidity, flexibility, and almost “interchangeability” among team members. In other words, there was a focus on what needed to be done, rather than on strict boundaries between roles, *i.e.*, “that is your job and this is my job”. Thus, staff were working together in a manner that aligned with their training, ethics and value systems. In this way, Facility A staff appeared to be more care-focused than task-focused which presented a win-win situation for staff in terms of job satisfaction and morale, and for residents in terms of quality of life.

### 4.2. Comparative Insights from Facility B

In terms of creating a climate for change at Facility B, while staff sensed that there may have been a larger vision guiding external leadership in designing the CDM, Facility B leaders were perceived as simply reacting to, rather than actively shaping the model implementation. When asked about how much information or education was provided in advance of the CDM, one participant expressed concern that staff experienced the model as a top-down, initiative in which they had little input:

*It’s one thing to hear it. It’s another thing to live it. You’ve got RNs and you’ve got these LPNs and they’ve got the training. It sounds like it should all work*, *but I think where any of these pieces fall apart is around change- change management and culture change and how we facilitate that. And that people when they don’t feel that they have any control and that things are being dictated to them*; *the resistance is so big.*

*(Allied Health Worker*, *Facility B)*

From this transcript it was evident that the CDM implementation was experienced differently in Facility B, more as an imposition than an opportunity, with staff feeling a strong lack of control. A nurse leader spoke about how challenging she and other staff were finding their roles in the post-CDM implementation period when asked how the model affected team dynamics:

*I’ve seen a lot of negative feelings*, *and you know pitting one against the other. I think when I listen…we have had staff meetings and when I listen to the LPNs I do feel sad. They have their reasons. And I hear the care aides and then they say “well there are LPNs…” you know this back and forth*, *but looking at the big picture*, *yeah I would do things differently.*

*…People do come and my role has changed. So back again I have to go to the manager and ask okay*, *what should we do now with this thing? And then you finish and then again another group and then the care aides are feeling frustrated…*

*…They’re looking for a team leader and people think residential*, *it’s not like acute*, *but you do need a good strong leader*, *and if you don’t build that it does affect the quality of care.*

*(Nurse Leader*, *Facility B)*

In terms of engaging and enabling the whole organization to work towards the change, it is notable that staff relations at Facility B were more fractious, with staff groups stacked against each other rather than aligned and working collaboratively. The Nurse Leader (above) also vocalized her opinion that large-scale changes such as this one require strong leaders. It is difficult to say if this comment was a reflection of the shorter time that leadership members had been in place at Facility B, or related to specific qualities or skills of the current leadership.

While there was awareness of the need to increase and create opportunities for camaraderie and collaboration, somehow a sense of there being no time for this was conveyed. In contrast to Facility A, research team interviewers were struck by the atmosphere of “dysfunction” and “discord” that seemed to pervade the environment in Facility B:

*[It’s] not because the staff have no compassion*, *no wish to learn. It’s that they’ve reached their limits. They need to put a hold and just look at each local site [and ask the question] How can we actually get the morale up again so people can have laughter and fun and have a potluck….You know staff would actually have some time connecting and ease off the stress they get from complaints from the family and the residents because they feel that we have each other.*

*(Manager*, *Facility B)*

At Facility B, the RCC was said to view her role within a context of rigid boundaries and clear responsibilities. Rather than helping on the floor directly, she maintained that her primary focus was to do the job that she was obligated to do within the context of her organizational role. By seeing her new role from a perspective of narrow boundaries instead of opportunities, and in failing to actively mentor and foster an environment of support and collaboration among staff, she jeopardized team cohesion.

The lack of a sense of control and feeling that the model was imposed from “above” were compounded in Facility B by the movement of LPNs through the single certification initiative. In this facility, the larger degree of staff turnover and greater reliance on part-time staff meant that leadership was forced to spend more time on recruitment, orientation, and education for new staff, instead of working with existing staff who were also in need of additional training and mentoring given their expanded, or altered scopes of practice, and ways of working together as a result of the CDM.

*Well*, *over the years we knew eventually that RNs would be replaced by LPNs*, *but the way it was done it wasn’t…I guess they did tell us it was coming*, *but it was just it was more of a shock when it first happened because it just…You’ve got all these young LPNs and inexperienced young LPNs working with acute and sub-acute people….if you don’t have the experience you can’t see what you need to see. You’re not sure what you’re observing*; *where once you have experience*, *and it takes years or at least working in a place if you work in an acute care facility you’re picking up all --- and you’re working with RNs*, *and you’re working with all different types of people. Where here the LPNs are working on their own and I don’t think they get what they need. There’s not the mentoring.*

*(LPN*, *Facility B)*

While efforts were certainly being made to promote communication at Facility B, concerns about scope of practice loomed large even for those staff with longstanding experience. Rather than breaking down barriers and enhancing team functioning, the CDM implementation seemed to create further mistrust, in-fighting and divisiveness among staff. Though many LPNs were considered to be operating at a high level in terms of their expertise, there was concern that they were working beyond their capacity, with little oversight on their tasks and without adequate mentoring, potentially compromising the care of residents. In essence, it appeared that Facility B staff were stuck in the “neutral zone” with a broad range of anxieties, stresses and worries about their jobs, and a concern about how the atmosphere had changed for the worse. In part, a “culture of resistance” to the model seemed to have been established, much of it coming from long-time employees such as HCAs who had been comfortable working with RNs in a particular way. As LPNs were given more responsibility, despite their experience, HCAs felt that their knowledge and experience was not as well recognized or regarded.

*And you know*, *I was happy coming to work all the time. I really enjoyed my work. I really did. I still like my job. I just don’t like the atmosphere anymore. The atmosphere has changed here so much. We feel like everything has been put on our shoulders and there’s people right down on you all the time*, *instead of you know like we used to laugh and joke a lot more. We still do*, *but not like we used to.*

*(HCA*, *Facility B)*

As noted, changes after model implementation were felt and tensions were high among care team members with concern expressed about the lack of staff helping other staff at Facility B.

*What they say to them [RNs] is oh if you have time you can come up and help on the floor. Well*, *all they do is just close that door and sit in there. I mean*, *and you’re too busy. The RN doesn’t give a crap because she’s off trying to hide*, *so she doesn’t have to do anything.*

*(HCA*, *Facility B)*

In terms of the third phase of implementing and sustaining change related to the CDM implementation, while leadership wanted to support staff and were concerned about their sense of lack of control and low morale, it seemed they also felt overwhelmed, lacking either the motivation, or the skills or knowledge to approach implementation from a more creative, empowering, side-by-side and collaborative perspective. As a consequence, the sense of oppressiveness and helplessness were amplified, and CDM implementation was experienced as stressful for everyone from the ranks of leadership through to direct care staff.

## 5. Discussion and Conclusions

We began this paper with the primary research question: “how did leadership style and approach influence team functioning with the implementation of a care delivery model in the residential care sector?” Our analysis of the data represent the main study findings. In this final section we consider the lessons learned from the implementation of the CDM locally and the broader implications of these findings for leadership under conditions of change management. This is followed by the identification of study limitations, and some final thoughts on health care leadership.

### 5.1. Lessons from the CDM

From the findings shared here it is evident that leadership style had a profound impact on CDM implementation. Ultimately, Facility A, experienced a more optimal transition and implementation process as a function of prior conditions, and a leadership style that was compassionate, empowering, collegial and supportive. At Facility B on the other hand, the CDM was experienced as disruptive, and onerous, and as a directive imposed upon facility staff and leadership with little consultation. Consequently, resistance was high and buy-in low, and morale and team functioning suffered.

Berwick [36] writes, “in innovation, new concepts usually must come from outside the current system, but new processes—the things that make the concepts live—must come from inside or they will not work” (p. 1974). In short, the *how* and process of CDM implementation operated very differently at Facility A and B; one looking at the model as an opportunity, and the other resisting more than embracing the CDM [24]. How the change initiative was positioned, *i.e.*, as “opportunity” *vs*. as “an imposition” on an already strained system had different consequences for team functioning and morale.

Some of the challenges for Facility B were aggravated by greater staff turnover. Primarily LPNs, but also some HCAs relocated for a host of reasons: to secure better shifts and more permanent positions, and for maternity leaves and retirements which increased reliance on part-time staff. With greater staff mobility and change, the leadership had less knowledge of their staff to mobilize existing skill sets, utilize expertise and build cohesion. To complicate matters further, there was more turnover among leadership team members at Facility B as well.

For Facility A staff turnover was minimal and human resource shifts (*i.e.*, increasing LPNs and decreasing RNs), had been addressed through the slower lead-up process to implementation, and from a servant leadership approach that encouraged “thinking outside the box”. Team members were supported each step of the way and encouraged to consider their hopes, dreams and roles for the future. This fostered high team functioning and aligns with the culture change literature in emphasizing multi-level participation and collaboration [20,28,30]. In this way Facility A leaders promoted a “how can we make this work?”, attitude that reduced anxiety and promoted job satisfaction and security, and sparked innovation, encouraging workers to perform at a higher level [29,31]. Managers were then able to work with existing strengths, capacities, and limitations to build strategies that were feasible, as well as supportive and empowering when enacted.

Conversely, at Facility B staff felt that the model had been developed as yet another top-down initiative, imposed in a non-consultative way, with little to no connection to their work-place realities. Further, they did not have a common vision to draw upon to be able to see the benefits of what they were being asked to do [24,28]. Consequently, staff were caught up in a cycle of powerlessness and lack of control, and morale deteriorated [22,23]. Leadership at Facility B expressed concern and awareness about the poor atmosphere, and low morale of staff, but felt they had little manoeuverability or opportunity for ingenuity with how the CDM was implemented [12,13]. In a separate interview with the CDM developer after the facility level data were collected, the research team was told that when the model was introduced at a higher level, middle managers (*i.e.*, facility level) were encouraged to take staff mix and composition into greater account based on their knowledge of existing dynamics and team functioning at their own facility. It is possible that this vision was not transmitted or enacted by local facility leadership. In any case, the leadership at Facility B struggled to implement the CDM for a host of reasons, notably the other concurrent initiatives underway (*i.e.*, a new resident assessment tool, and single certification) which taken together, served to overwhelm them.

Two other related points are worth noting in regards to the lessons from the CDM. These have both narrow and broader meaning in the context of this study and can be subtitled: *It’s the little things that count*, and *it’s the people and not the model*. For the former, having a safe space for open and inclusive dialogue to occur was invaluable for staff at Facility A. At Facility B, however, staff and leadership felt stretched to the limit, and opportunities to interact with one another on a personal or social level had been curtailed to the detriment of relationship-building, or the development of a collaborative culture [16,17,18,19,20]. While both facilities were operating under conditions of shrinking financial resources, staff at Facility A strategized about opportunities for staff-resident engagement, e.g., undertaking potlucks and staff and resident recognition events. They fundraised through organized bake sales and hamburger days to continue to host such events. A key message for all levels of leaders here is that small-scale events such as this may have immeasurable benefits for empowering staff and promoting the development of good relationships and good relations between staff and residents [37,38].

In the case of “it’s the people, not the model”, one illustration of this was the different attitudes of the RCCs at each facility. RCCS occupy a pivotal, bridging position between facility management and direct care staff. They can facilitate relationship-building and prevent problems from becoming large and immobilizing to the extent that they can exacerbate team dysfunction. At Facility A, the RCC had a pitch in and help attitude, while at Facility B, the RCC had a rigid, narrow view of her role and it did not entail much in the way of mentoring or support. Further, Facility A had two RCCs with higher levels of leadership training and different attitudes than were found in Facility B. Such capacity may have allowed facility A to attend to care differently, while in Facility B, the lack of additional RCC capacity, or sufficient capacity in general for the number of residents, was also complicated by having more part-time RNs. This mix of staff and issues may have contributed to reduced team cohesion, and a deeper sense of burden and overload. Ultimately, it’s the people who comprise the team and who matter in positive or negative terms through the power of specific roles and functions such as RCCs, or through the power of team members working together.

### 5.2. Broader Lessons for Leadership under Change Management

The CDM was designed as a model of service delivery to address issues of equity in service provision. Here, equity could be looked at as a technical problem, or as an adaptive challenge, but may be more appropriately viewed as the latter given its complexity and the lack of simple singular solutions by which it can be addressed [25]. Returning to the original goals of the CDM; setting the standard of 3.0 h of care per resident, standardizing the funding methodology between subsidized and non-subsidized facilities, and changing the staff mix ratio for direct care staff, may be viewed as technical solutions to the more challenging adaptive problem of caring for vulnerable older adults in an equitable way. While providing three hours of care to each resident addresses issues of equality; addressing equity in care might suggest the need to provide different levels of service to each resident based on their specific needs and vulnerabilities [16,18,29]. Thus, equity issues are more complex and multi-layered and tied to the above named goals, but also to questions about quality of care [36,38].

In the research context section, we presented two styles of leadership more or less in opposition to each other; servant and transactional. In practice leadership styles are often a blend of many different approaches [24,25,26,27] that do not always fit into neat and tidy boxes. While Facility A exemplified servant leadership to a strong degree, Facility B leadership exhibited tendencies towards transactional, hierarchical relationships from managers down to direct care staff. At the same time, management exhibited concern and compassion for staff at both facilities, which is a common feature of servant leadership.

Nembhard and Edmondson [39] describe an entrenched hierarchy as one that is “based on status and the relative power, prominence, respect and influence” that shapes health care practice (p. 944). They emphasize that “levelling the playing field”, or creating side-by-side relationships rather than hierarchical ones, can enable more effective and inclusive inter-professional engagement, and can ultimately have a positive impact on the generation of ideas, buy-in for projects, and the development of a sense of safety between practitioners, as was evident at Facility A. In the longer term, effective leaders can foster more stability among their teams if objectives for care are underlain by common value systems, and under conditions that offer safety, support and empowerment [5,16,29,36,37,38,39]. Thus, health care leaders need skills related to policy, human resource practices, and operational protocols, but should also possess, or be able to develop skills related to leading teams through periods of complex, transformative change [6,21,24]. The propensity for ongoing transformation in the health care environment make it imperative to invest in the development of effective leaders [15,16,17,18,19,20].

In servant leadership support for relationship-building and a strong sense of community are critical. Everything that happens *or doesn’t happen* in a facility reflects something about how the organization works, and what (and who), is valued [11,21,22]. At Facility B, antecedent challenges and conditions, and the lack of consultation around implementation sharply limited “relational integration” and opportunities for team cohesion and capacity-building. This “community” value must be seen as large and encompassing, suffusing *all* of the interpersonal relationships in any particular health care or residential care setting, rather than a select few. In other words, if quality of care rests on a foundation of solid, supportive, empowering relationships [12], then efforts to improve quality of care must at some level be based on improving all relationships as well, be they between leadership and direct care staff, between staff and staff, or between staff and residents and family members [36].

At another level, implementation processes around new care delivery models may fail to consider how relationships between staff (and residents) are damaged or disrupted at a very basic, face-to-face level by changing roles and job mobility that alter team composition and practice. When such innovations arise, staff need their leadership to understand their need to adjust, recalibrate and even grieve the loss of old ways of operating, and also the loss of staff members from among their ranks, even as they are working to move out of the neutral zone towards new beginnings [22,23].

In the context of multiple, concurrent changes, it is also relevant to ask, “How much change is too much change?” As noted, having sufficient lead time and a slower progression leading up to large, new change initiatives and innovations, allows leadership and inter-professional teams to better prepare for the changes that are coming. Further, despite ongoing human resource and financial constraints in the health care system, leadership needs to work with staff at all stages to develop a collective vision for how models will unfold. The absence of an explicit vision that can evolve and be shared, contributes to the sense of fear, mistrust, lack of control and overload that can characterize staff relationships during times of change. Decision-makers working at a higher level from middle management (facility level) need to do what they can to protect facilities, direct care staff, residents and their families, as well as the local leadership from facing too much change and immediate adaptation [22,23].

### 5.3. Limitations and Final Thoughts

In this study, at least two limitations are identified. Not surprisingly, the first limitation was that it was difficult to separate the impact of the CDM from other simultaneous changes that were underway and this made it challenging to fully isolate how leadership influenced the CDM uptake. Second, the same staff and leadership could not be interviewed at both time points (summer 2012 and 2013), in particular in Facility B, due to staff turnover and scheduling, whereas at Facility A, leadership was interviewed twice because turnover was low. If this had not been the case, it would have been possible to compare the two facilities along this dimension of change over time.

Trastek *et al.* [24] and Heifetz and Linsky [27] maintain that sometimes a leader has to admit they do not have all the answers, or do not know for sure how something will turn out. What they need, however, is an environment that supports their creativity and willingness to reach out and try different strategies, knowing that the team is strong enough to innovate and succeed, or to innovate and fail. In either case, important lessons will be learned. Corazzini *et al.* [28] invoke the principles of “complexity theory” to remind us that leadership is an emergent property of health care organizational systems and must therefore be viewed as dynamic and evolving rather than as unchanging and stagnating.

In the years ahead health and caregiving organizations must create the conditions (*i.e.*, strong leadership and team functioning, good communication, and the development and translation of common visions), that allow members (*i.e.*, staff) to move *towards each other*, rather than *away from each other* during times of stress and anxiety [40]. This type of environment was exemplified at Facility A. At Facility B not only did practitioners move away from each other, they also seemed to build larger walls at times that prevented cohesiveness and strong team functioning.

Findings from the current study offer contrasting images of a care delivery model innovation. We conclude that the CDM was experienced very differently at the two residential care facilities, and that these unique experiences were attributable in large measure to variations in leadership styles and approaches, and also to other important factors such as previous historical context, degree of planning and preparation in advance of the implementation, nature of staff (skills, mix and numbers), number and nature of concurrent initiatives, and the speed, magnitude and direction of each new care delivery initiative. Servant styles of leadership have the potential to help health care system managers and directors to adapt to health system challenges and client complexity. In an environment that is constantly evolving and changing, leaders must proactively work with teams to establish the direction and create the conditions to help staff move forward in a direction that promotes quality of life for both residents and workers [34,35].

As Heifetz *et al.* [30] state “People don’t resist change, they resist loss” (p. 11). For example, HCAs expressed their deep concern over the loss of RNs at Facility B. At these times, leaders must be adept at reading the signs, diagnosing and responding to perceptions of loss, and considering the implications that change has for individuals [25]. Under the right conditions, a servant leadership style can support a range of positive outcomes like growth, access to jobs, wealth, status, community, loyalty and identity ([25] p. 10). In the end, as well as developing a vision, creating common values, inspiring growth, modeling change, advancing skills and building competencies; good leadership is also a quality of the heart [27], to be approached with openness, wisdom, and compassion in the service of those who receive as well as provide care.
